# Robust probabilistic superposition and comparison of protein structures

**DOI:** 10.1186/1471-2105-11-363

**Published:** 2010-07-01

**Authors:** Martin Mechelke, Michael Habeck

**Affiliations:** 1 Department of Protein Evolution, Max-Planck-Institute for Developmental Biology, Spemannstr. 35, 72076 Tübingen, Germany; 2 Department of Empirical Inference, Max-Planck-Institute for Biological Cybernetics, Spemannstr. 38, 72076 Tübingen, Germany

## Abstract

**Background:**

Protein structure comparison is a central issue in structural bioinformatics. The standard dissimilarity measure for protein structures is the root mean square deviation (RMSD) of representative atom positions such as α-carbons. To evaluate the RMSD the structures under comparison must be superimposed optimally so as to minimize the RMSD. How to evaluate optimal fits becomes a matter of debate, if the structures contain regions which differ largely - a situation encountered in NMR ensembles and proteins undergoing large-scale conformational transitions.

**Results:**

We present a probabilistic method for robust superposition and comparison of protein structures. Our method aims to identify the largest structurally invariant core. To do so, we model non-rigid displacements in protein structures with outlier-tolerant probability distributions. These distributions exhibit heavier tails than the Gaussian distribution underlying standard RMSD minimization and thus accommodate highly divergent structural regions. The drawback is that under a heavy-tailed model analytical expressions for the optimal superposition no longer exist. To circumvent this problem we work with a scale mixture representation, which implies a weighted RMSD. We develop two iterative procedures, an Expectation Maximization algorithm and a Gibbs sampler, to estimate the local weights, the optimal superposition, and the parameters of the heavy-tailed distribution. Applications demonstrate that heavy-tailed models capture differences between structures undergoing substantial conformational changes and can be used to assess the precision of NMR structures. By comparing Bayes factors we can automatically choose the most adequate model. Therefore our method is parameter-free.

**Conclusions:**

Heavy-tailed distributions are well-suited to describe large-scale conformational differences in protein structures. A scale mixture representation facilitates the fitting of these distributions and enables outlier-tolerant superposition.

## Background

Conformational heterogeneity is a common theme in protein structures and relevant in a wide range of different contexts. Proteins are flexible macromolecules whose function is often accompanied by a structural transition [1,2]. Allostery, for example, is a ubiquitous mechanism in signal transduction [3] and continues to be a controversial field of research [4]. Structural heterogeneity may also stem from a lack of data. NMR structures are usually represented as ensembles of conformers that fit the data equally well [5]. Here structural heterogeneity mainly reflects a scarcity of restraints and not necessarily true conformational flexibility.

When comparing protein structures in different conformational states, one is mainly interested in internal structural changes rather than differences that can be accounted for by a rigid-body movement. The separation of external from internal movements directly relates to the problem of how to compare and superimpose protein structures in different conformational states.

The hallmark of protein structure comparison is the root mean square deviation (RMSD) between equivalent atom positions after the rigid modes of structural change have been removed. The RMSD defines an optimality criterion to determine the rotation and translation that best separate rigid-body from internal movements. How to minimize the RMSD over all possible translations and rotations is a classical problem in structural bioinformatics and has been treated by many authors [6]. After early accounts by Diamond and McLachlan [7,8], Kabsch has given a closed analytical expression for the optimal translation and rotation in terms of a singular value decomposition [9]. Kearsley and others have provided an alternative solution based on quaternions that improves the Kabsch algorithm in terms of speed and stability [10,11].

A physical justification for superimposing protein structures by RMSD minimization originates in the theory of dynamics in semi-rigid molecules. Eckart has derived [12] conditions for the separation of external (rotational and translational) from internal modes of movement, if the molecule is subject to small-amplitude vibrational motions. Recently, it has been pointed out that structure comparison by RMSD minimization is equivalent to searching for the frame of reference that satisfies Eckart's conditions [13,14]. Therefore, if one considers a set of heterogenous structures an ensemble of fluctuating states, RMSD minimization is the physically correct method for removing rigid-body displacements. However, in many situations one is interested in a frame of reference that is different from the Eckart frame. Such a situation may occur if we want to compare proteins that undergo structural transitions upon interaction with other molecules. This is a non-equilibrium situation in which the protein is driven to a different energy basin. A classical example is adenylate kinase comprising three rigid domains that undergo an opening-closing conformational transition upon the binding of substrate [15]. Here, RMSD fitting fails to highlight the relative rigid-body movements leading to domain closure. Because the assumption of vibrational conformational changes is not fulfilled over the entire polypeptide chain, atoms that belong to the mobile domains would appear as "outliers" that cannot be described by vibrational dynamics.

RMSD minimization is a least-squares technique and therefore suffers from the same problems that least-squares methods have in other data analysis applications, namely sensitivity to outliers. Problems with outliers in protein structure comparison have been treated in a number of ways. One simple fix is to extend the Kabsch formula to weighted RMSD superposition. A weight is assigned to every atom and applied when summing over the distances between equivalent positions. By choosing small weights for outliers their dominance can be alleviated. Other approaches build on different metrics than Euclidean distances. Lesk [16], for example, uses the Chebyshev distance to identify common substructures in proteins. The LMS fit algorithm [17] minimizes the median rather the mean squared deviation between equivalent atoms. Another class of methods seeks to find a flexible, rather than a rigid alignment between protein structures [18].

The possibility to weight atoms individually is already mentioned in Kabsch's paper but no rule for setting the weights is given. As a consequence, different schemes for weighting atoms have been proposed. An important class of algorithms iteratively filters out atoms whose deviation exceeds a predefined threshold [19-23]. This strategy tries to identify those atoms that make up an invariant structural core. More recent applications assign continuous weights to every atom, such that all atoms contribute to the weighted RMSD. The Gaussian-weighted RMSD (wRMSD) method [24] updates the weights iteratively by plugging distances between equivalent positions into a Gaussian distribution. Wu and Wu [25] set the weight to a theoretical temperature factor predicted with a Gaussian network model.

Here we elaborate on the idea of using a weighted RMSD to find a superposition that identifies geometrically similar substructures in heterogeneous protein structures. The method is intended for the comparison of structures that exhibit significant structural disparity or undergo large-scale conformational change. We introduce probabilistic models that describe very generic properties of the large-amplitude structural changes that we want to account for. We show that learning these models solves the superposition problem also in the presence of gross structural transitions and is equivalent to a weighted RMSD whose weights are updated iteratively. A problem with existing methods minimizing a weighted RMSD is that the choice of the weights is heuristic and often depends on user-defined parameters such as thresholds [19,23] and decay constants [24,25]. Our approach is principled and provides objective rules for setting the weights. By averaging analytically over the weights we show that our models for structural displacements are heavy-tailed distributions, which are often used to describe extreme events, for example, in economics. Model comparison techniques allow us to choose among the different models and objectively select the one that is most supported by the structures. We illustrate the applicability of the approach in various contexts ranging from structural changes in proteins to NMR ensembles. From a practical point of view, the new method provides an objective and robust basis for protein structure comparison and superposition.

## Results and Discussion

### Frames of reference in protein structure comparison

**Figure 1:**
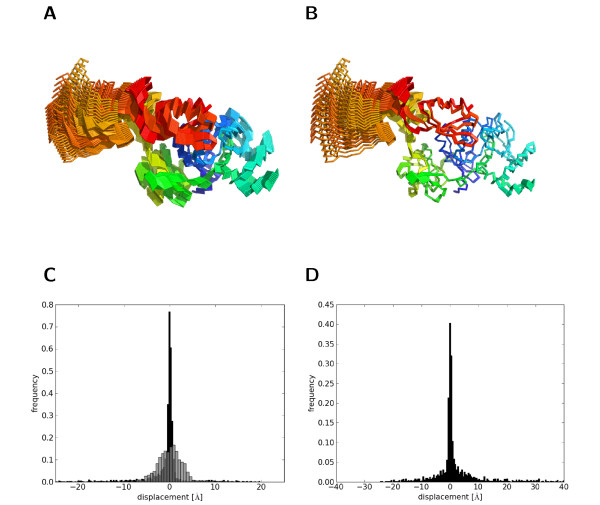
**Structural changes viewed from different frames of reference**. A: Conformations of elongation factor G sampled along the first principal component. Conformations are optimally superimposed according to RMSD because they are generated in the Eckart frame. B: The core-weighted fit aims to superimpose the structurally invariant part, irrespective of the underlying physical mechanism. Viewed from this frame of reference most of the changes happen in the C-terminal domains (domain IV and V). C: Empirical distribution of the displacements in the Eckart frame (grey) and according to the core-weighted fit (black). D: Distribution of the displacements relating the bound and unbound structure of GroEL.

Rather than adopting a coordinate system prescribed by physical principles such as the Eckart frame, we are interested in finding a frame of reference that overlays heterogenous protein structures such that they share a tight, maximally large structural core at the cost of large-scale outliers. To illustrate the difference consider a normal-mode simulation of Elongation factor G (PDB code 1FNM), which contains five compact domains. We used the elastic network model implemented in MMTK [26] to sample conformations about 1FNM. The elastic network model imposes purely vibrational interactions between restrained atoms. Snapshots along the first principal mode are, by construction, related only via internal displacements. This is indeed verified by calculating the RMSD between, for example, the first and last conformation: the optimal rotation is the identity matrix, the translation vector zero. In the weighted RMSD frame that maximizes the overlay of spatially invariant positions, the differences between the two structures are interpreted as a finite rigid-body movement (Figure 1A, B).

To describe this situation quantitatively, we assume that alternative positions of the *i*th atom are encoded by three-dimensional vectors **x**_*i *_and **y**_*i*_. The most general relation between equivalent positions is given by the generative model:

where the rigid-body transformation involves a rotation matrix **R **and translation vector **t **and the vectors **d**_*i *_are the non-rigid displacements. In the Eckart frame, the structures are displaced around the center of mass, whereas in a superposition maximizing the common structural core only domain IV seems to move (see Figure 1A, B). Consequently, we observe different distributions for the displacements depending on whether we superimpose by unweighted or core-weighted RMSD. In the weighted fit, the distribution of displacements exhibits a narrow central peak corresponding to the well-fitting core and broad tails accounting for large-amplitude movements (Figure 1C). In the Eckart frame, the displacements are distributed more homogeneously. We observe similar large-amplitude displacements in a comparison of GroEL in ATP-free and ATP-bound state (Figure 1D) demonstrating that the shape of the distribution is universal irrespective of the specific context.

If we knew the displacements exactly, we could obtain the rotation and translation by solving the above system of equations. In general, however, the internal displacements are unknown and need to be estimated simultaneously with the superposition. This is a chicken-and-egg problem: estimation of the rigid body transformation requires knowledge of the internal changes, which themselves can be calculated only if the superposition is known. In general, the decomposition into rigid-body and internal changes is not unique. We need additional principles to estimate the decomposition from a set of heterogenous structures.

### Modeling non-rigid structural change in proteins

To separate internal from external structural changes we need to make assumptions about their properties. Our assumptions will be of statistical nature, because we do not know the correct displacements and can only infer them from the given structures. We encode our assumptions in a probability distribution over the displacement vectors *f*(**d**). Given this distribution, a statistical approach to protein structure comparison proceeds by plugging the displacements calculated according to generative model into the distribution. This results in the total likelihood for the separation into rigid and non-rigid structural changes under the assumed model *f*(**d**):

and depends on the choice of the rigid-body transformation. The optimal separation is obtained by maximizing *L*(**R, t**) over all rotations and translations to implicitly obtain displacements whose distribution best matches *f*(**d**).

What are reasonable assumptions about non-rigid structural changes in proteins that are realistic and, at the same time, simple enough to allow for efficient computation? One straightforward property of *f*(**d**) is that its mean is zero, because if it were not, the mean could not be distinguished from the overall translation. Second, it should not matter which of the two structures we superimpose onto the other. The displacements resulting from either superposition should follow the same distribution. The reverse of the generative model is **x**_*i *_= **R**^*T*^**y**_*i *_- **t **- **R**^*T*^**d**_*i*_. That is,  = -**R**^*T*^**d**_*i*_ are the displacements according to the reverse superposition expressed through the parameters of the original superposition. We demand *f*() = *f*(**d**_*i*_) from which follows that *f*(**d**_*i*_) is isotropic, i.e. it depends only on the norm of the displacements, not their direction.

An intuitive quantity to characterize the displacements are their expected amplitudes *a*_*i *_which are the second moments *f*. Because we assume *f *to be isotropic, we only need to consider the average squared norm of the displacements  rather than the full covariance matrix. For a Gaussian model, the amplitude is directly related to the isotropic variance *σ*^2^: *a*_*i *_= 3*σ*^2^. The assumption underlying RMSD fitting is that the unknown displacements occur in a homogenous fashion: all amplitudes vary on the same scale *σ*, which is a statistical analog of the Eckart assumption. However, as discussed before, we are interested in modeling displacements that are most of the time small but occasionally huge.

A natural extension is to associate a separate variance  with each displacement. For mathematical convenience, we work with precisions *s*_*i*_ =  rather than variances; *s*_*i *_can be viewed as a measure of local stiffiness. By allowing the *s*_*i *_to adopt values of diverse orders of magnitude, we can model displacements that vary on different scales. According to this model, highly restrained atoms will be assigned large *s*_*i *_as is the case, for example, for core atoms that are restricted in their mobility by nearest neighbor interactions. On the opposite end of the spectrum, large-scale displacements as, for example, in T7 RNA polymerase [2] would be described with *s*_*i*_'s that are close to zero. Under a multi-scale model of structural change, the optimal rotation and translation is obtained by maximizing the corresponding total likelihood or rather minimizing its negative logarithm:

where the inverse amplitudes *s*_*i *_=  are atom specific weights.

### Amplitude spectra of large-scale conformational changes

If the amplitudes of the displacements were known, the optimal rigid body transformation could be determined by a weighted superposition. However, the scales *s*_*i *_are unknown, and it seems that we just shifted the problem. We need to estimate the scales and therefore must make assumptions about their distribution *g*(*s*). Because the scales are non-zero, their distribution should only have support on the positive axis. If we interpret the *s*_*i *_as force constants, the spectrum should span a wide range of variability owing to the fact that protein structures are subject to internal forces of different strengths. We use a Gamma and an Inverse Gamma distribution for *g*(*s*). These distributions are flexible enough to gradually switch between different situations in which we have more or less homogeneously distributed amplitudes. The functional form of Gamma and Inverse Gamma distributions is governed by two parameters, a shape parameter *α *and a scale *β*, which is an overall scale of the displacement amplitudes.

**Figure 2:**
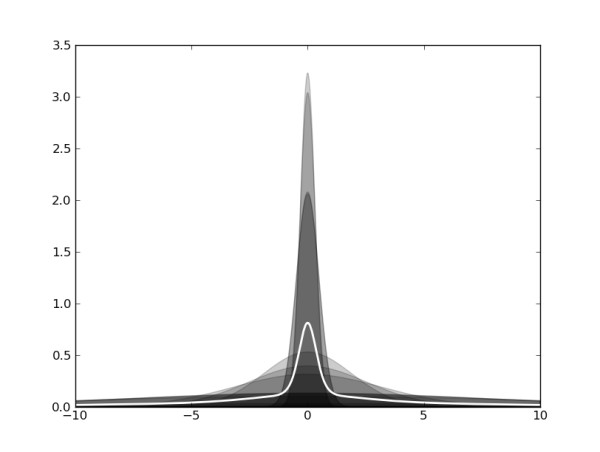
**Modeling non-rigid displacements by scale mixtures**. Our model for the distribution of displacement vectors is a mixture of isotropic Gaussians with increasing width (grey filled curves). By averaging of the Gaussians, we obtain a heavy-tailed effective distribution (white line) that exhibits a narrow central peak and broad tails. By adding more and more components one achieves in the limit of infinitely many components an exact representation of the heavy-tailed distribution. In this limit, the average is obtained as an integral over Gaussians of continuously varying width. How the widths are distributed is governed by a density function *g*(*s*) of the scales or inverse widths *s*. The functional form of the mixing distribution and of the implied heavy-tailed model is govern by the shape and scale parameters *α *and *β*.

What is the distribution of the displacements *f*(**d**) implied by our choice of *g*(*s*)? To answer this question we need to average over all scales. Figure 2 illustrates this averaging process. The effective distribution of non-rigid displacements exhibits a dominant central peak but also allows outliers to occur occasionally, which is reflected in the elevated "fatness" of the tails when compared with a Gaussian distribution. In the statistical literature, distributions of this form are called heavy-tailed. Heavy-tailed distributions are robust against outliers in data [27] and can often be represented as averages over Gaussian distributions with zero mean and increasing width, so-called scale mixtures of Gaussian distributions [28,29]. From a pragmatic point of view, the average distribution of internal displacements has the convenient property that it is heavy-tailed and thereby accommodates large-amplitude structural changes. For a Gamma and Inverse Gamma-distribution, the effective distribution *f*(**d**) can be calculated analytically. The former corresponds to a Student *t *distribution, the latter is a member of the *K *distribution family [30] comprising the Laplace distribution as a special case. In their superposition algorithm THESEUS [31], Theobald and Wuttke use a related model. They introduce a multivariate Gaussian with a full-covariance structure for inter-positional dependencies and estimate the covariance matrix during the superposition. The eigenvalues of the covariance matrix are assumed to be distributed according to an Inverse Gamma distribution. In case of a diagonal covariance matrix, the model is equivalent to a Gamma prior on the scales (inverse variances) and thus to the Student *t *model.

### Algorithms for disentangling rigid and non-rigid structural changes

The generalized optimization problem that we face when maximizing *L*(**R**, **r**) is to minimize:

That is, *f*(**d**) implies the generalized metric - log *f*(**d**) for comparing equivalent atom positions. For Gaussian *f*, this metric is the squared Euclidean distance. For Laplace-distributed displacements, it is the Euclidean distance (not its square). Analytical expressions for the best rigid transformation under such metrics are not known. One could optimize the negative log-likelihood numerically. However, we will pursue an alternative approach based on the scale mixture representations that we discussed in the previous section.

**Figure 3:**
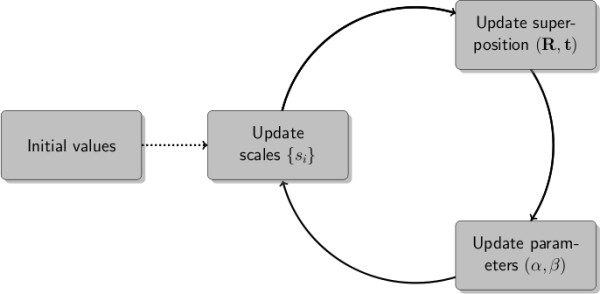
**Iterative disentanglement of rigid and non-rigid displacements**. Flowchart showing the iterative estimation of atom specific weights (scales), the rigid-body transformation and the parameters of the distribution of the scales. The initial scales are generated randomly. Both EM and Gibbs sampling operate by cycling through the updates until convergence is reach. The EM updates are deterministic, the Gibbs sampling updates stochastic.

The basic idea is illustrated in a flowchart (Figure 3). Instead of averaging analytically over the scales, we estimate them simultaneously with the rigid transformation and the parameters *α *and *β *that determine the shape of *g*(*s*). The algorithm proceeds iteratively by updating the different groups of parameters, {*s*_*i*_}, (**R**, **t**) and (*α*, *β*), separately. Updates of the rigid transformation involve a weighted RMSD fit. How to update the weights *s*_*i*_, the shape *α *and the scale *β *is detailed in the Methods section and additional file 1.

We have developed two version of the updates: a deterministic and a stochastic algorithm. The latter, a Gibbs sampler, allows us to estimate not only the optimal parameters, but also their uncertainty and enables comparison of different models, as will be shown later. Both algorithms converge very rapidly within the first 50 iterations, after which the likelihood does no longer improve significantly. On a modern computer the best EM fit is obtained within a few fractions of a second. The Gibbs sampler is approximately ten times slower. Running times for both algorithms on different protein structure pairs are provided in additional file 1.

### Robust fitting of proteins subject to large conformational changes

**Figure 4:**
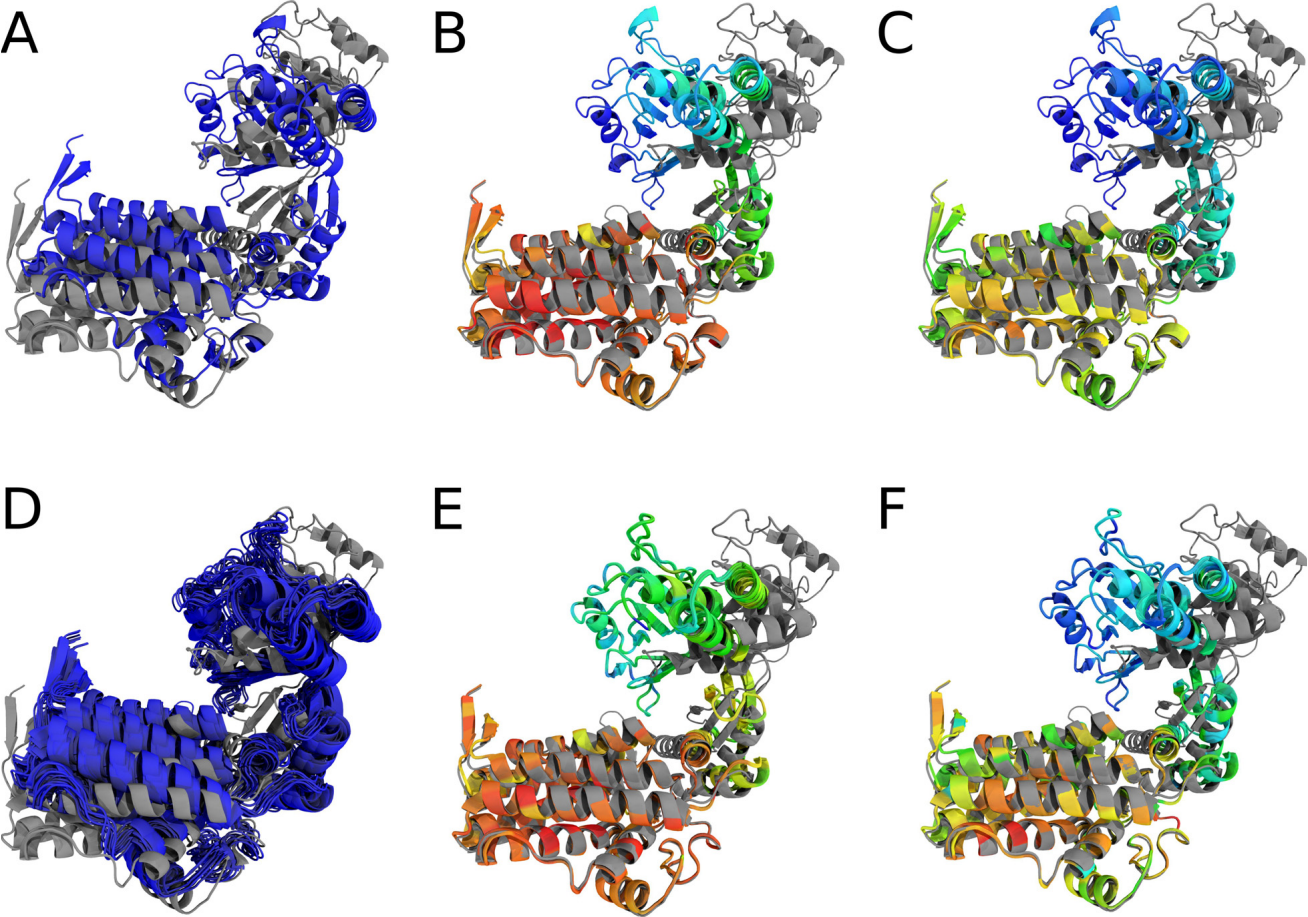
**Superposition of bound and unbound GroEL**. Cartoon representation of the superposition of the bound and unbound state of GroEL (1AON (grey) and 1OEL (colored)) using a Gaussian model (A, D), a Student *t *(B, E) model and a *K *distribution (C, F). Large local scales are shown in red, whereas blue indicates small weights. The upper row (A-C) depicts the results of an EM superposition. The lower row (D-F) shows the ensembles of 25 orientations generated by the Gibbs sampling procedure. The ensembles obtained for the Student *t *and *K *distribution show little variance as opposed to the ensemble obtained with a Gaussian model.

**Figure 5:**
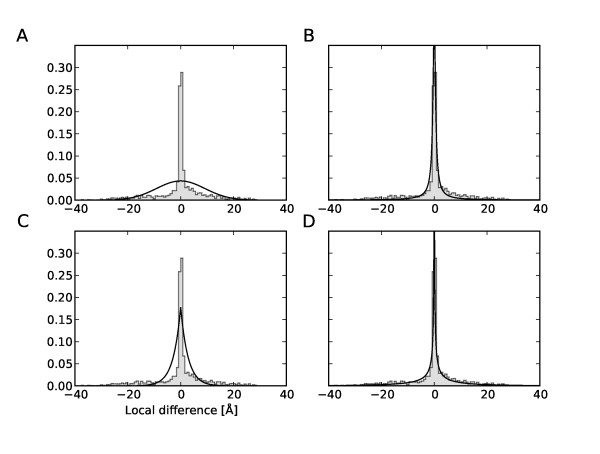
**Distribution of local deviations between conformational states of GroEL**. Pooled distribution of local structural differences in all three spatial directions between the bound and unbound state of GroEL (1AON and 1OEL) is shown as grey histogram. Black solid lines are fits of a Gaussian (A), Student *t *(B), Laplace (C) and *K *distribution (D).

**Figure 6:**
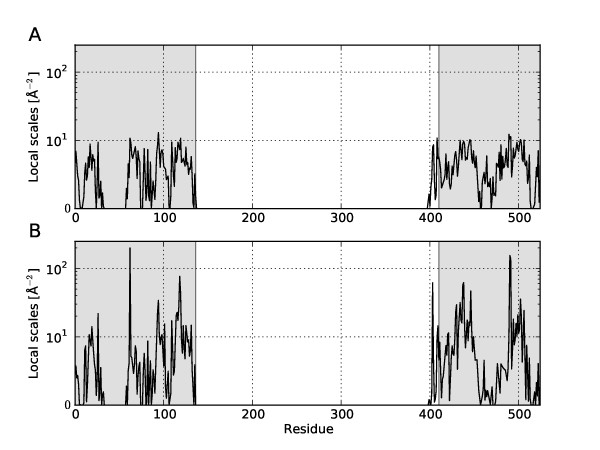
**Local scales and domain structure in GroEL**. The black solid line are the traces of the local scales according to the Student *t *(A) and *K *distribution (B) obtained in an analysis of bound and unbound structures of GroEL. The grey shaded regions highlight the equatorial domain (1-136, 410-526), which remains conformationally invariant during the transition from the open to the closed conformation. The intermediate domain spans regions 137-190 and 367-409, the apical domain comprises residues 191-366.

To demonstrate the validity of our approach, we tested the framework on a number of proteins undergoing large conformational changes. Figure 4 shows the superposition of two different conformations of GroEL [32,33] based on a Gaussian, Student *t*, and *K *distribution. The transition of GroEL from an unbound to a bound state involves rigid body movements of the intermediate and apical domains. The superposition based on a Gaussian model fails to reveal the relative movements of the domains, whereas the heavy-tailed models converge on a tight fit of the equatorial domain. This observation is supported by an analysis of the individual domains. Although the overall RMSD increases from 12.3 Å for the least-squares superposition to 15.6 Å for both non-Gaussian methods, the RMSD of the equatorial domain drops from 7.4 Å down to 1.5 Å and 1.3 Å for the Student *t *and *K *distribution, respectively. Both values are close to the optimal RMSD of 1.2 Å when fitting the equatorial domain alone. The reliability of the superposition can be assessed through the structure ensembles obtained by applying the random transformations generated during Gibbs sampling. The ensemble generated by Gaussian superposition is broad reflecting a high degree of uncertainty. In contrast, the ensembles based on heavy-tailed distributions are narrow, which indicates that the superposition is very well defined (Figure 4). The findings are confirmed by looking at the histograms of non-rigid displacements and their parametric fits (Figure 5). Only the heavy-tailed distributions fit the displacments reasonably well, whereas a Gaussian fails to describe the simultaneous occurance of many well-fitting positions and a few large-scale outliers. These fits are obtained automatically during the superposition - once a model has been chosen, all unknown parameters are estimated self-consistently (how to choose a model is explained below). In contrast, other superposition algorithms based on a weighted RMSD involve adjustable parameters that are set heuristically. This may lead to problems with identifying the optimal structural core (see additional file 1 for an example). The domain architecture of GroEL is highlighted by a trace plot of the local scales (Figure 6). Large weights are assigned to the well fitting equatorial domain and almost all weights for the intermediate and apical domain are close to zero.

**Figure 7:**
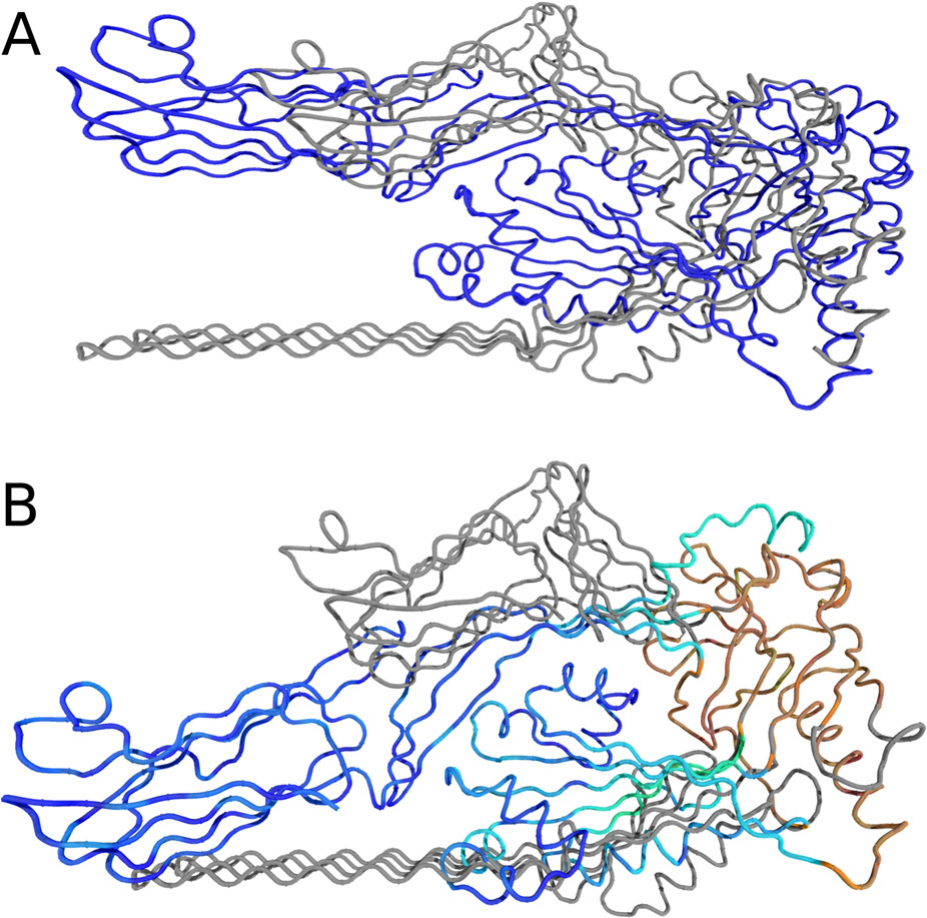
**Superpositions of Pneumolysin**. Estimated superposition of Pneumolysin (PDB codes 2BK1 (grey) and 2BK2 (colored)) using a Gaussian (A) and Student *t *(B) model. The superposition based on the *K *distribution is visually not distinguishable from the one obtained with a Student *t *distribution and thus not shown. Large local scales are shown in red whereas blue indicates small weights.

Heavy-tailed models are well-suited to describe even large conformational changes. This is exemplified for Pneumolysin [34] which, upon membrane insertion, refolds two of its four domains leading to an invariant core of about 30% of all residues only. Figure 7 compares the least-squares superposition with superpositions based on a Student *t *and *K *distribution. Again only a non-Gaussian superposition is able to locate and fit the invariant region.

### Superposition of NMR ensembles

NMR structures are usually represented as ensembles that reflect the quality and completeness of the data as well as the local precision of the structure [5,35]. Often termini and loops show high variability either due to protein dynamics or missing data. If one wants to assess the precision of an NMR structure, superposition by RMSD minimization often fails to reflect local differences due to variations in restraint density [23,31]. As a consequence, no generally accepted way to fit ensembles exist. The superposition is often determined based on secondary structure elements or subjective criteria such as a small number of manually defined positions. Our framework provides a more objective, robust and model-driven alternative to such practice.

**Figure 8:**
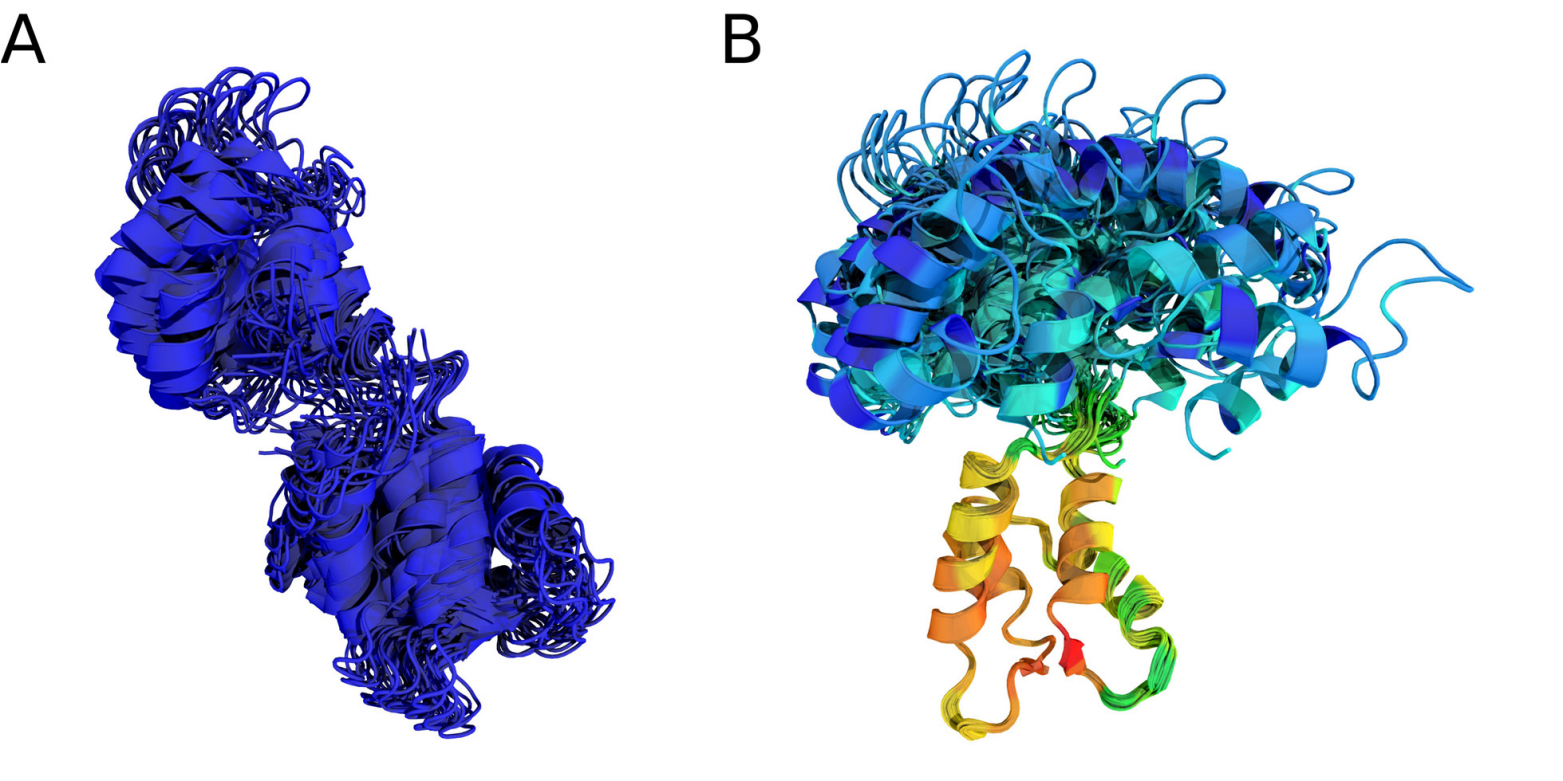
**Superposition of an NMR ensemble of Calmodulin**. All conformations are superimposed onto the estimated average structure by the EM algorithm using a Gaussian (A) and a Student *t *(B) model. The superposition according to the *K *distribution is visually not distinguishable from the one of the Student *t *distribution and thus not shown. Large local scales are shown in red, whereas blue indicates small weights.

A particularly suited example to demonstrate the violation of the least-squares assumption is the NMR ensemble of Calmodulin (PDB code 1CFC). Calmodulin is involved in cellular regulation and consists of two identical domains connected by a flexible hinge region [36]. The flexibility is also reflected in the NMR ensemble. If one domain is superimposed, the other undergoes a large relative motion, which makes a joint superposition impossible. Figure 8 highlights differences between a Gaussian and an outlier-tolerant superposition for this example. The heavy-tailed models favor a superposition onto the N-terminal domain with an internal RMSD of 0.7 Å, whereas the least-squares algorithm fails to find a tight superposition. By tweaking the initial weights, it is possible to find the superposition onto the C-terminal domain with an RMSD of 1.7 Å, albeit this superposition has a lower likelihood. The higher likelihood of a superposition onto the N-terminal domain is consistent with the observation by Kuboniwa et al. [36] that the N-terminal is better defined than C-terminal domain and shows a lower internal RMSD.

### Bayesian model comparison

So far, we a given model of the displacements to a set of structures. Bayesian inference allows us to go further and infer which model is the most appropriate given a set of structures. This information is provided by the *evidence *or *marginal likelihood P*(*M*|*D*), the probability of model *M *(Gaussian, Student *t*, or *K *distribution) given data *D *(the structures under comparison in our context) [37]. The evidence is computed by integration over all possible parameter values. Two models *M*_1 _and *M*_2 _are ranked relative to each other through the Bayes factor *P*(*M*_1_|*D*)/*P *(*M*_2_|*D*) [38,39]. If the Bayes factor is significantly greater than one, the data favor model *M*_1 _over model *M*_2 _and vice versa. Here we seek to assess whether to choose the Student *t *or *K *distribution over a Gaussian model for structure superposition and comparison. Because the evidence is not amenable to analytical evaluation, we use an estimator calculated from the posterior samples obtained with Gibbs sampling [40].

Table 1 lists the estimated log-evidence for nine structure pairs obtained for a Gauss, Laplace, Student *t *and *K *model. In all cases, the heavy-tailed models (Student *t *and *K *distribution) are significantly better supported by the data than the Laplace and the Gauss distribution. For GroEL and pneumolysin the *K *distribution is preferred over a Student *t *model which agrees well with the visual impression obtained from the projected distributions shown in Figure 5. In all other instances the Student *t *model seems to provide the best description of the internal displacements. We also ran tests on synthetic data generated according to the Student *t *and the Gaussian model (see last two rows of Table 1). In case of Student *t *distributed displacements, the Student *t *model achieves the highest marginal likelihood closely followed by the *K *distribution. This shows that the model selection by comparing estimated Bayes factors works. The Laplace and Gauss distribution models have significantly lower evidence because they are not flexible enough to accomodate large-amplitude displacements. In case of Gaussian distributed displacements, none of the four alternative models is really preferred over the others. The marginal likelihood values are identical within the precision of the estimation procedure. This is reasonable because scale mixtures are nested models and include the Gaussian distribution as a limiting case. The test demonstrates that heavy-tailed models can cope with purely Gaussian displacements equally well as the standard RMSD and are also suitable to analyse rigid displacements.

**Table 1:** Marginal likelihood of different models

Protein	PDB IDs	Student *t*	*K*	Laplace	Gauss
GroEL	1AON-1OEL	-4328.57	**-4307.22**	-5132.84	-5722.35
DNA Pol	1IH7-1IG9	**-5574.80**	-5750.12	-6340.01	-8011.05
RAN	1RRP-1BYU	**-1124.86**	-1176.53	-1795.92	-2286.10
Topo II	1BGW-1BJT	**-4496.17**	-4553.50	-7210.74	-8042.00
Pneumolysin	2BK2-2BK1	-2692.73	**-2465.09**	-5195.85	-5491.90
ER	3ERD-3ERT	**-538.11**	-622.69	-1290.81	-1980.96
RNA Pol	1QLN-1MSW	**-5296.61**	-5455.79	-8471.69	-10168.07
Adenylate Kinase	1AKE-4AKE	**-1499.73**	-1502.75	-1685.11	-2000.35
Myosin	1B7T-1DFK	**-4819.91**	-5046.02	-6380.11	-7701.83


Synthetic data	Student *t*	-9179.46	-9253.48	-12465.97	-13951.94
Synthetic data	Gauss	-5108.73	-5112.43	-5077.97	-5115.98

Logarithm of the marginal likelihood *P*(*M*|*D*) of the different displacement models obtained for nine structure pairs undergoing domain movements. Highlighted in boldface are the maximum log-marginal likelihood values. The last two rows at the bottom report the log-marginal likelihoods for synthetic data generated according to a Student *t *and a Gauss distribution.

## Conclusions

We present a robust probabilistic approach to protein structure superposition and comparison. The approach builds on heavy-tailed distributions to model non-rigid displacements between protein structures. To estimate these distributions and an optimal superposition we employ a scale mixture representation of the heavy-tailed models. Practically, this amounts to introducing weights for each atom position and to estimate the weights iteratively during structure superposition. In contrast to other weight-based superposition methods, the scale mixture framework provides a firm statistical basis for setting the weights. Moreover, the link to the closed form helps to interpret the weighting scheme in terms of heavy-tailed models for structural displacements.

## Methods

### Scale mixture representation of heavy-tailed distributions

We use a scale mixture of Gaussian distributions [28,29]

to represent the distribution *f*(**d**) of the displacement vectors **d **between protein structures under comparison. *N*(**d**; **0**, *s*^-1^) is the zero-centered, isotropic Gaussian distribution in three-dimensional space with variance *s*^-1^·*g*(*s*) is the prior distribution of the inverse variances or scales *s*. If we choose a Gamma distribution as mixing density *g*(*s*), we obtain the three-dimensional Student *t *distribution:

where *α *is a shape parameter and *β *a scale. If we use the Inverse Gamma distribution for *g*(*s*), we obtain the three-dimensional *K *distribution:

where again *α *determines the shape of the distribution and *β *the (inverse) scale; *K*_*v *_is the modified Bessel function of the second kind. For the special case *α *= 2 and *a *=  we recover the Laplace distribution:

1 D projections of these scale mixtures are used to visualize the agreement between the empirical distribution of conformational displacements (see Figure 5).

### Parameter estimation

The EM algorithm [41] and the Gibbs sampler [42] are iterative algorithms that estimate the rigid transformation and the functional form of the heavy-tailed distribution with the help of the auxiliary variables *s*_*i*_. The main difference is that EM is a deterministic algorithm that calculates a single point estimate, whereas Gibbs sampling is a stochastic method that generates a posterior sample. The Gibbs sampler samples from a joint distribution by repeatedly replacing a randomly chosen variable by a sample from its distribution conditioned on the remaining variables. Upon convergence the samples generated by the Gibbs sampler follow the joint distribution of interest. The benefit of Gibbs sampling over EM is that it calculates not only a point estimate but a posterior sample that can be used to estimate parameters by posterior means and evaluate parameter uncertainties as posterior variances. Moreover, the posterior sample can be used to estimate the evidence of the model given the data.

#### Updates of the scales

The positional scales *s*_*i *_can be learned from their conditional posterior distribution *N*(**d**_*i*_; **0**, ) *g*(*s*_*i*_; *α*, *β*). For the Student *t *model, the mixing distribution *g*(*s*; *α*, *β*) is a Gamma distribution *G*(*s*; *α*, *β*). The conditional posterior of *s*_*i *_is also a Gamma distribution *G*(*s*_*i*_; *α *+ 3/2, *β *+ ||*d*_*i*_||^2^/2). For the *K *distribution, the mixing density is the Inverse Gamma distribution *IG*(*s*; *α*, *β*). The conditional posterior of *s*_*i *_is the Generalized Inverse Gaussian distribution [43]* GIG*(*s*_*i*_; 3*/*2 - α, ||*d*_*i*_||^2^, 2*β*). In the E-step of the EM algorithm, we replace the scales by their expectation values under the Gamma and the GIG distribution, respectively. The analytical expression for the expectation values are given in additional file 1. During Gibbs sampling, we update the scale by generating a random sample from the Gamma and the GIG distribution (see additional file 1).

#### Estimation of the rigid transformation

In the M-step of the EM algorithm, the optimal rotation and translation are determined by minimizing a weighted RMSD in which the local scales *s*_*i *_are positional weights. During Gibbs sampling, the translation is generated from an isotropic three-dimensional Gaussian distribution. The rotation is sampled from the conditional posterior of functional from exp {tr (**A**^*T*^**R**)} with . How random rotations can be generated from this distribution is described in [44].

#### Estimation of model parameters

For every set of protein structures under comparison the optimal model parameters *α *and *β *will be different. Therefore we need to treat them as unknown variables and estimate them case by case. For both the Student *t *and the *K *distribution, the conditional posterior distribution of the scale parameter *β *is a Gamma distribution. Analytical expressions for the expectation values are used in the M-step of the EM algorithm. The Gibbs sampler, draws random variates from  (Student *t *distribution) or from  (*K *distribution), respectively. Inference of the shape parameter *α *is more involved.

Under both heavy-tailed models *α *cannot be maximized analytically. Therefore we need numerical optimization methods to update this parameter. In EM, we employ a root finding method to maximize the logarithm of the conditional posterior probability of *α*. During Gibbs sampling, we use adaptive rejection sampling [45], a technique to generate random variates from an arbitrary log-concave distribution. To achieve a fully probabilistic treatment and to avoid numerical instabilities, we further assume a Gamma distributed hyperprior for *α *and *β*.

### Evaluation of the marginal likelihood

Bayesian model comparison ranks alternative models according to their evidence or marginal likelihood. In our application, calculation of the evidence involves the integral:

where *D *are the structures under comparison (the data) and *M *a model for the distribution of conformational differences (i.e. Gaussian, Student *t*, or generalized Laplace). *π*(*α*, *β*) denotes the hyperprior on the parameters of the heavy-tailed distribution (Gamma distributions), *π*(**R**) a uniform prior distribution over rotations and *π*(**t**) a broad Gaussian prior over the translations centered at zero.

*L*_*M *_(**R, t**, *α*, *β*) is the likelihood function implied by the current model (i.e. for *M *being the Student *t *distribution *L *is the total product of 3 D Student *t *densities). Given samples from the joint posterior distribution *π*(*α*, *β*) *π*(**R**) *π*(**t**) *L*_*M *_(**R, t**, *α*, *β*) (using Gibbs sampling and the scale mixture trick), we evaluate the marginal likelihood by applying the harmonic mean estimator proposed by Newton and Raftery [40].

### Software

The algorithm has been implemented in the scripting language Python and is publically available at http://toolkit.tuebingen.mpg.de/bfit.

## Authors' contributions

MH designed research. MM carried out research and analyzed data. MM and MH wrote the manuscript. The authors read and approved the final manuscript.

## Supplementary Material

Additional file 1 **Supporting information**. The additional file contains tables reporting the running times of EM and Gibbs sampling for the different heavy-tailed models. For comparison, running times of WRMSD [24] are also listed. A comparison of the rotation found with our method and WRMSD is provided for the nine structure pairs listed in Table 1. An example where WRMSD gives a supoptimal superposition is shown. Further details on the algorithm are described. Click here for file
