# Supplementary material for: Robust probabilistic superposition and comparison of protein structures
Source: BMC Bioinformatics. 2010 Jul 1;11:363. doi: 10.1186/1471-2105-11-363 (PMC2912885; doi:10.1186/1471-2105-11-363)
Supplement: Additional file 1 — Supporting information. The additional file contains tables reporting the running times of EM and Gibbs sampling for the different heavy-tailed models. For comparison, running times of WRMSD [24] are also listed. A comparison of the rotation found with our method and WRMSD is provided for the nine structure pairs listed in Table 1. An example where WRMSD gives a supoptimal superposition is shown. Further details on the algorithm are described. [file 1471-2105-11-363-S1.PDF]

Supporting Material: Robust probabilistic superposition and comparison of protein structures

Martin Mechelke[1] and Michael Habeck[1,2,]

March 17, 2010

(1) Department of Protein Evolution, Max-Planck-Institute for Developmental Biology, Spemannstr. 35, 72076 Tübingen, Germany
(2) Department of Empirical Inference, Max-Planck-Institute for Biological Cybernetics, Spemannstr. 38, 72076 Tübingen, Germany

Contents

1 Tables reporting running times and parameter estimates 2
1.1 Parameter estimates 2
1.2 Running times EM 3
1.3 Running times Gibbs Sampler 3

2 Comparison with Gaussian-weighted RMSD (wRMSD) 4

3 Scale mixtures of Gaussians 5

4 Expectation maximization and Gibbs sampling 6
4.1 Expectation Maximization 6
4.2 Gibbs sampling 7
4.3 Estimation of the scales 7
4.4 Estimation of the rigid transformation 8
4.5 Estimation of the parameters of the heavy-tailed models 9
4.6 Initialization 9
4.7 Superposition of structure ensembles 10
4.8 Generating random variates from the Generalized Inverse Gaussian distribution 10

1 Tables reporting running times and parameter estimates

Table 1 reports parameter estimates and errors obtained with the Gibbs sampler on the nine structure pairs also analysed in Table 1 of the main article. The last two tables provide the running times of our models in the EM (Tab. 2) and Gibbs sampling (Tab. 3) mode. For comparison running times obtained with the Gaussian-weighted RMSD (wRMSD) method [1] are also provided. The wRMSD method updates the weights iteratively by plugging distances between equivalent positions into a Gaussian distribution with a heuristically chosen width. The test set comprises the same nine structure pairs as before. The wRMSD method was run until a convergence criterion was reached. For our probabilistic algorithms we performed 50 optimization/sampling steps, which in all cases were sufficient to reach convergence.

1.1 Parameter estimates

Proteins	K-distribution		Student t distribution	
	α	β	α	β
1BGW-1BJT	0.1612 ± 0.0001	0.0007 ± 0.0000	0.2163 ± 0.0001	0.0146 ± 0.0000
1B7T-1DFK	0.2350 ± 0.0001	0.0044 ± 0.0000	0.4405 ± 0.0008	0.2589 ± 0.0014
1QLN-1MSW	0.1656 ± 0.0000	0.0011 ± 0.0000	0.2481 ± 0.0002	0.0215 ± 0.0000
1IH7-1IG9	0.4209 ± 0.0002	0.0393 ± 0.0000	0.6487 ± 0.0015	0.5702 ± 0.0039
1AON-1OEL	0.2636 ± 0.0002	0.0031 ± 0.0000	0.2687 ± 0.0002	0.1192 ± 0.0006
1AKE-4AKE	0.3735 ± 0.0012	0.0205 ± 0.0000	0.3687 ± 0.0020	0.1814 ± 0.0035
2BK1-2BK2	0.1372 ± 0.0000	0.0004 ± 0.0000	0.0756 ± 0.0000	0.0001 ± 0.0000
1RRP-1BYU	0.1795 ± 0.0002	0.0028 ± 0.0000	0.4128 ± 0.0011	0.0956 ± 0.0004
3ERD-3ERT	0.2016 ± 0.0003	0.0413 ± 0.0001	0.4874 ± 0.0024	0.0165 ± 0.0000

Table 1: Estimates of the parameters describing the shape and scale of the heavy-tailed models.

1.2 Running times EM

Algorithm<br>Protein	Student-t<br>Time $[s]$	K<br>Time $[s]$	Gauss<br>Time $[s]$	wRMSD<br>Time $[s]$
1BGW-1BJT	0.2593	0.2572	0.0002	58.4982
1B7T-1DFK	0.2676	0.2658	0.0002	9.6844
1QLN-1MSW	0.3043	0.3016	0.0002	6.1786
1IH7-1IG9	0.3318	0.3287	0.0003	19.9579
1AON-1OEL	0.2243	0.2227	0.0002	60.3066
1AKE-4AKE	0.1408	0.1402	0.0002	1.6952
2BK2-2BK1	0.1996	0.1986	0.0002	58.9352
1RRP-1BYU	0.1321	0.1317	0.0002	1.0888
3ERD-3ERT	0.0870	0.0877	0.0002	2.1010
average	0.2163	0.2149	0.0002	24.2717

Table 2: Running times of EM

1.3 Running times Gibbs Sampler

Algorithm<br>Protein	Student-t<br>Time $[s]$	K<br>Time $[s]$	Gauss<br>Time $[s]$	wRMSD<br>Time $[s]$
1BGW-1BJT	1.9646	9.1303	0.03064	58.4982
1B7T-1DFK	1.3440	9.2768	0.03254	9.6844
1QLN-1MSW	2.0378	9.3161	0.03344	6.1786
1IH7-1IG9	1.3957	4.1522	0.03264	19.9579
1AON-1OEL	1.9100	9.1979	0.03044	60.3066
1AKE-4AKE	1.4715	8.3887	0.02534	1.6952
2BK2-2BK1	1.7831	7.5843	0.02444	58.9352
1RRP-1BYU	1.5211	8.7280	0.02414	1.0888
3ERD-3ERT	1.1449	8.8593	0.02354	2.1010
average	1.6191	8.2926	0.02857	24.2717

Table 3: Running times of the Gibbs sampler

2 Comparison with Gaussian-weighted RMSD (wRMSD)

We compared the rotation found with the Student t and K distribution with the optimal rotation according to wRMSD. Figure 1 shows the three Euler angles found by the different methods.

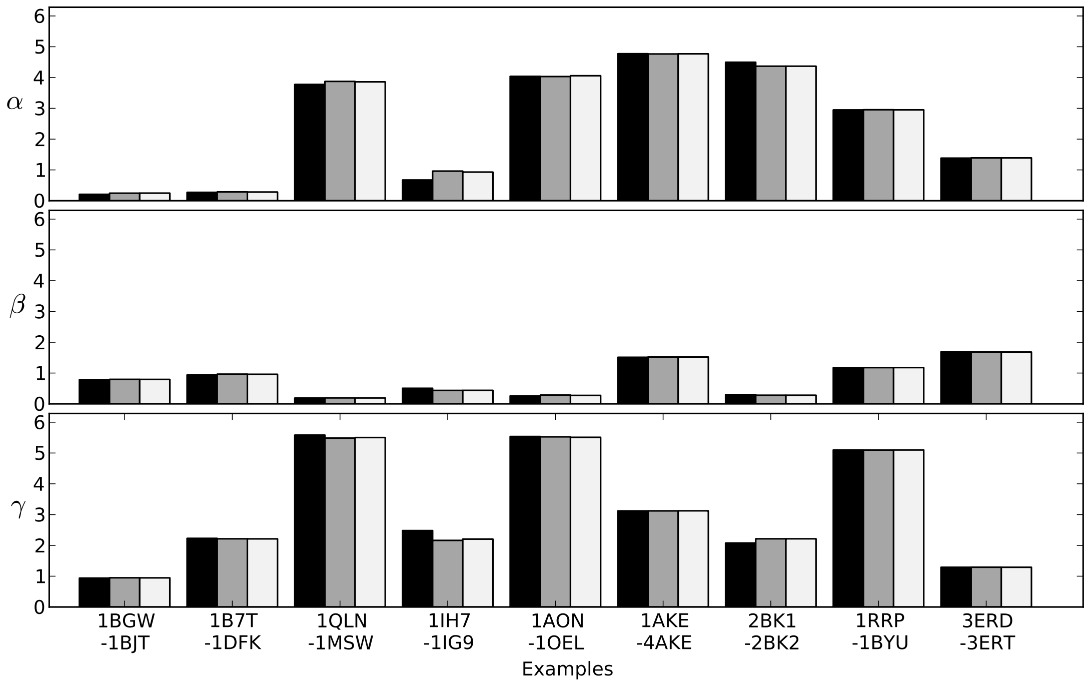

Figure 1: Euler angles (α, β, γ) of the rotation matrices generated with wRMSD (black bars) and a heavy-tailed fit based on the Student t distribution (grey bars) and on the K distribution (light grey bars).

The Euler angles differ for DNA polymerase (example 4) and Pneumolysin (example 7). These differences translate to the superpositions shown in Figure 2 for DNA polymerase. wRMSD identifies only a subset of the conformationally invariant core, which can be attributed to an improper heuristic estimate of the scaling factor used in Gaussian weights. In contrast, our method finds the invariant core between the two conformational states. A similar situation is encountered in Pneumolysin where wRMSD fails to identify the core region that can be superimposed perfectly. If the wRMSD scaling factor is optimized manually, it is possible to recover the same superposition as reported by our algorithm.

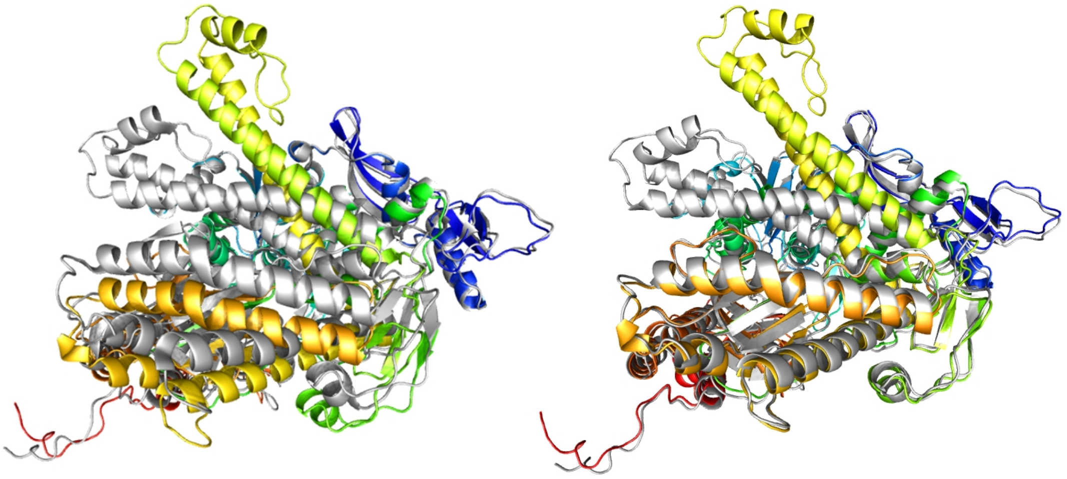

Figure 2: Superposition of DNA Polymerase (PDB codes 1IG9 (grey) and 1IH7 (colored)) according to wRMSD (left) and the Student t model (right).

3 Scale mixtures of Gaussians

Formally, the scale mixture representation of a heavy-tailed model $f(\mathbf{d}; \alpha, \beta)$ for the distribution of the three-dimensional difference vector $\mathbf{d}$ reads:

$$f(\mathbf{d}; \alpha, \beta) = \int \mathrm{d}s \; N(\mathbf{d}; \mathbf{0}, s^{-1}) \, g(s; \alpha, \beta) = \int \mathrm{d}s \, (s/2\pi)^{3/2} e^{-\frac{s}{2}\|\mathbf{d}\|^2} \, g(s; \alpha, \beta). \quad (1)$$

The choice of the scale prior distribution $g(s; \alpha, \beta)$ determines the functional form of the non-Gaussian distribution. If we choose a Gamma distribution as mixing density $g(s)$, we obtain the three-dimensional Student t distribution:

$$\begin{aligned} f_{\text{Student}}(\mathbf{d}; \alpha, \beta) &= \int \mathrm{d}s \; (s/2\pi)^{3/2} \, e^{-\frac{s}{2}\|\mathbf{d}\|^2} \, \frac{\beta^\alpha}{\Gamma(\alpha)} \, s^{\alpha-1} e^{-\beta s} \\ &= (2\pi\beta)^{-3/2} \frac{\Gamma(\alpha + 3/2)}{\Gamma(\alpha)} \left[1 + \|\mathbf{d}\|^2/2\beta\right]^{-(\alpha+3/2)} \end{aligned}$$

where α is a shape parameter and β a scale. If we use the Inverse Gamma distribution for $g(s)$, we obtain the three-dimensional K distribution:

$$\begin{aligned} f_{\text{K}}(\mathbf{d}; \alpha, \beta) &= \int \mathrm{d}s \; (s/2\pi)^{3/2} \, e^{-\frac{s}{2}\|\mathbf{d}\|^2} \, \frac{\beta^\alpha}{\Gamma(\alpha)} \, s^{-(\alpha+1)} e^{-\beta/s} \\ &= \frac{(\sqrt{2\beta})^{\alpha+3/2}}{2^{\alpha-1}(2\pi)^{3/2}\Gamma(\alpha)} \|\mathbf{d}\|^{\alpha-3/2} \, K_{3/2-\alpha}(\sqrt{2\beta}\|\mathbf{d}\|) \end{aligned}$$

where again α determines the shape of the distribution and β the (inverse) scale; K_ν is the modified Bessel function of the second kind. For the special case $\alpha = 2$

and $a = \sqrt{2\beta}$, we recover the Laplace distribution:

$$f_{\mathrm{Laplace}}(\mathbf{d}; a) = \frac{1}{8\pi}\, a^3 e^{-a\|\mathbf{d}\|}. \tag{2}$$

1D projections of the scale mixtures are used to visualize the agreement between the empirical distribution of conformational displacements. Because the projection of a three-dimensional Gaussian is a one-dimensional Gaussian, the projected scale mixture will also be a scale mixture:

$$f(d_k) = \int \mathrm{d}s\, N(d_k; 0, s^{-1})\, g(s); \quad k = x, y, z \tag{3}$$

where d_k denotes the (signed) deviation in x-, y-, and z-direction (for example, $d_x = \mathbf{e}_x^T(\mathbf{x} - \mathbf{y})$ where $\mathbf{e}_x$ is the unit direction vector pointing along the x axis). That is, one-dimensional projections of the three-dimensional Student t distributions are one-dimensional Student t distributions. The same holds for the family of K distributions, however for the special case of a Laplace distribution the projected density will not be a 1D Laplacian but a more general one-dimensional K distribution.

4 Expectation maximization and Gibbs sampling

We propose two closely related algorithms to update the scales and to estimate the rotation, translation and model parameters α and β. In the Expectation Maximization (EM) [2] framework, the optimal scales are the conditional expectation values for given $\mathbf{R}$, $\mathbf{t}$, and α, β. The parameters of primary interest, $\mathbf{R}$, $\mathbf{t}$, and α, β, are estimated by optimization. The Gibbs sampler [3] updates the scales by generating random samples from their conditional posterior distribution for given $\mathbf{R}$, $\mathbf{t}$, and α, β. Parameters of primary interest are updated by drawing posterior samples conditioned on the current scales. The EM algorithm only provides a point estimate of the parameters of interest. The Gibbs sampler, on the other hand, explores the full posterior distribution and thereby also allows the quantification of parameter uncertainties.

4.1 Expectation Maximization

The EM method treats the scales s_i as missing data and cycles between updates of the scales (E-step) and updates of the model parameters $(\mathbf{R}, \mathbf{t})$ and (α, β) (M-step). Formally, we introduce the extended log-likelihood function of observed and missing data:

$$-\log L(\mathbf{R}, \mathbf{t}, \alpha, \beta, \{s_i\}) = \frac{1}{2} \sum_i s_i \|\mathbf{y}_i - \mathbf{R}\,\mathbf{x}_i - \mathbf{t}\|^2 - \log g(s_i; \alpha, \beta) \tag{4}$$

The E-step calculates the *average* log-likelihood function:

$$-\langle \log L(\mathbf{R}, \mathbf{t}, \alpha, \beta)\rangle = \frac{1}{2}\sum_i \langle s_i\rangle \|\mathbf{y}_i - \mathbf{R}\,\mathbf{x}_i - \mathbf{t}\|^2 - \langle \log g(s_i; \alpha, \beta)\rangle \tag{5}$$

which involves calculation of the expected weights $\langle s_i\rangle$ for updating the rigid transformation and calculation of $\langle s_i^{-1}\rangle$ and $\langle \log s_i\rangle$ for updating the parameters of the heavy-tailed distribution.

4.2 Gibbs sampling

In Gibbs sampling we cycle through the following sampling steps:

$$\begin{aligned}
s_i &\sim s_i^{3/2} e^{-\frac{s_i}{2}\|\mathbf{d}_i\|^2} g(s_i; \alpha, \beta) && (6)\\
\mathbf{t} &\sim N(\mathbf{t}; \bar{\mathbf{t}}, (\nu + n\bar{s})^{-1}) && (7)\\
\mathbf{R} &\sim \exp\Big\{ \mathrm{tr}\Big[\mathbf{R} \sum_i s_i \mathbf{x}_i (\mathbf{y}_i - \mathbf{t})^T \Big] \Big\} && (8)\\
\beta &\sim G(\beta; n\alpha + \alpha_\beta, n\bar{s} + \beta_\beta) && (9)\\
\alpha &\sim \frac{(\hat{s}\beta)^{n\alpha}}{\Gamma(\alpha)^n} G(\alpha; \alpha_\alpha, \beta_\alpha) && (10)
\end{aligned}$$

Here "$\sim$" means "drawn from", in most cases the generation of random variates employs off-the-shelf random number generators. The above updates are valid for the Student t distribution. For the Laplace and K distribution the last two updates are slightly different. In the following, we provide details on the updates used in EM and Gibbs sampling.

4.3 Estimation of the scales

The basic quantity to estimate the scales in both EM and Gibbs sampling is the conditional posterior:

$$p(s_i|\mathbf{R}, \mathbf{t}, \alpha, \beta) \propto s_i^{3/2}\, e^{-\frac{s_i}{2}\|\mathbf{d}_i\|^2}\, g(s_i; \alpha, \beta) \tag{11}$$

where $\mathbf{d}_i$ is the difference between $\mathbf{x}_i$ and $\mathbf{y}_i$ after the transformation $\mathbf{R}, \mathbf{t}$ has been applied to $\mathbf{x}_i$. If $g(s_i; \alpha, \beta)$ is a Gamma distribution, the conditional posterior will also be a Gamma distribution:

$$p_{\mathrm{Student}}(s_i|\mathbf{R}, \mathbf{t}, \alpha, \beta) = G(s_i; \alpha + 3/2, \beta + \|\mathbf{d}_i\|^2/2) \propto s_i^{\alpha+1/2} e^{-(\beta + \|\mathbf{d}_i\|^2/2)s_i}. \tag{12}$$

During EM, the scales are updated using the expectation values:

$$\langle s_i\rangle = \frac{2\alpha + 3}{2\beta + \|\mathbf{d}_i\|^2}. \tag{13}$$

The Gibbs sampler generates a random variate from the Gamma distribution (12).

If $g(s_i;\alpha,\beta)$ is an Inverse Gamma distribution, the conditional posterior is a Generalized Inverse Gaussian distribution [4]:

$$p_{\mathrm{K}}(s_i|\mathbf{R},\mathbf{t},\alpha,\beta) = GIG(s_i; 3/2-\alpha, \|\mathbf{d}_i\|^2, 2\beta) \propto s_i^{1/2-\alpha} \exp\Big\{-\frac{\|\mathbf{d}_i\|^2 s_i + 2\beta/s_i}{2}\Big\}. \tag{14}$$

During EM, the scales are updated using the expectation values:

$$\langle s_i \rangle = \frac{\sqrt{2\beta}}{\|\mathbf{d}_i\|} \frac{K_{5/2-\alpha}\left(\sqrt{2\beta}\,\|\mathbf{d}_i\|\right)}{K_{3/2-\alpha}(\sqrt{2\beta}\,\|\mathbf{d}_i\|)}. \tag{15}$$

The Gibbs sampler generates a random variate from the Generalized Inverse Gaussian distribution in Eq. (14) (see supporting material). For the special case of a Laplacian ($\alpha = 2$, $\beta = a^2/2$) the conditional posterior is an Inverse Gaussian with expected scales:

$$\langle s_i \rangle = \frac{a}{\|\mathbf{d}_i\|} \tag{16}$$

4.4 Estimation of the rigid transformation

Given values for the positional scales s_i, the conditional posterior distribution of the translation vector is a three-dimensional isotropic Gaussian:

$$p(\mathbf{t}|\mathbf{R},\{s_i\}) = N(\mathbf{t};\bar{\mathbf{t}},(\nu+n\bar{s})^{-1}) \tag{17}$$

where ν is the inverse variance of a zero-centered three-dimensional hyperprior on $\mathbf{t}$ (typically $\nu = 10^{-3}$) and $\bar{s}$ the arithmetic mean of the scales. The conditional posterior is centered at $\bar{\mathbf{t}} = \frac{1}{\nu+n\bar{s}} \sum_i s_i(\mathbf{y}_i - \mathbf{R}\,\mathbf{x}_i)$. During EM, we update $\mathbf{t}$ by setting it to $\bar{\mathbf{t}}$. During Gibbs sampling, we generate a Gaussian variate from (17).

The conditional posterior distribution of the rotation matrix $\mathbf{R}$ is:

$$p(\mathbf{R}\,|\,\mathbf{t},\{s_i\}) \propto \exp\Big\{\mathrm{tr}\Big[\mathbf{R}\sum_i s_i\mathbf{x}_i(\mathbf{y}_i-\mathbf{t})^T\Big]\Big\} \tag{18}$$

We use the algorithm outlined in [5] to generate a random rotation from (18) during Gibbs sampling. For EM estimation, we need to carry out a weighted fit where the scales act as weights for the positions. This involves a singular value decomposition [6].

4.5 Estimation of the parameters of the heavy-tailed models

For both Student t and K distribution, the conditional posterior distribution of the scale parameter β is a Gamma distribution. In case of the Student t model, β follows $G(\beta; n\alpha + \alpha_\beta, n\overline{s} + \beta_\beta)$ where α_β and β_β are the shape and scale parameters of a Gamma hyperprior on β (both parameters are typically set to 10^{-3}). In case of the K distribution β follows $G(\beta; n\alpha + \alpha_\beta, n/\underline{s} + \beta_\beta)$ where $\underline{s}$ is the harmonic mean of the scales. In EM, $\overline{s}$ and $1/\underline{s}$ are the arithmetic averages of the expected scales and inverse scales. In the latter case (K distribution), we need to calculate:

$$\langle s_i^{-1} \rangle = \frac{\|\mathbf{d}_i\|}{\sqrt{2\beta}} \frac{K_{1/2-\alpha}\left(\sqrt{2\beta}\,\|\mathbf{d}_i\|\right)}{K_{3/2-\alpha}(\sqrt{2\beta}\,\|\mathbf{d}_i\|)} \tag{19}$$

under the GIG conditional posterior of the scales [Eq. (14)]. For the special case of the Laplace distribution, this simplifies to $\langle s_i^{-1} \rangle = \frac{\|\mathbf{d}_i\|}{a} + \frac{1}{a^2}$. In this case, the scale parameter ($a = \sqrt{2\beta}$) is updated using $a = 2\sqrt{n/\sum_i \langle s_i^{-1} \rangle}$.

The conditional posterior distribution of the shape parameter α is not of a standard form:

$$p(\alpha|\beta, \{s_i\}) \propto \frac{(\hat{s}\beta)^{n\alpha}}{\Gamma(\alpha)^n}\, G(\alpha; \alpha_\alpha, \beta_\alpha) \tag{20}$$

where $\hat{s}$ is the geometric mean of the scales and the right hand factor a Gamma-shaped hyperprior on α involving parameters α_α, β_α typically set to 10^{-3}. Eq. (20) holds for the Student t model, for the K distribution we have to replace the geometric mean by its reciprocal value. A convenient property of $p(\alpha|\cdot)$ is that it is log-concave. We can thus apply adaptive rejection sampling [7] to update this parameter during Gibbs sampling. In EM, we maximize the logarithm of $p(\alpha|\cdot)$ using a Newton algorithm similar to the one of Minka [8]. The objective function is obtained by calculating $\langle \log g(s_i; \alpha, \beta) \rangle$ where one averages over the local scales drawn from their conditional posterior. For the Student t model, this involves calculation of:

$$\langle \log s_i \rangle = \Psi(\alpha + 3/2) - \log(\beta + \|\mathbf{d}_i\|^2/2) \tag{21}$$

where $\Psi(x) = \mathrm{d} \log \Gamma(x)/\mathrm{d}x$ is the digamma function. In case of the K distribution, we have:

$$\langle \log s_i \rangle = -\log \frac{\|\mathbf{d}_i\|}{\sqrt{2\beta}} + \partial_\nu \log K_\nu(\sqrt{2\beta}\|\mathbf{d}_i\|)\Big|_{\nu=3/2-\alpha} \tag{22}$$

4.6 Initialization

Both EM algorithm and Gibbs sampler converge only locally. It might therefore be necessary to run multiple cycles from different starting positions to reach a

global optimum. We start with randomly generated scales followed by updates of the superposition and model parameters. Being iterative algorithms, it is further necessary to break off after a certain number of iterations is exceeded or a convergence criterion is met. In our experience, both algorithms converge very rapidly within 20 to 50 iterations and are stable against the choice of starting values.

4.7 Superposition of structure ensembles

The problem of superimposing two structures can be generalized to an ensemble of structures. Especially in case of large conformational heterogeneity, it seems inappropriate to choose one member of the ensemble as a reference onto which all other structures are transformed. Therefore we introduce an unknown average structure $\overline{\mathbf{Y}}$ that we need to estimate as well. We assume that all ensemble members can be superimposed onto $\overline{\mathbf{Y}}$ by individual transformations $\mathbf{R}_n$, $\mathbf{t}_n$. The model generalizes to

$$\overline{\mathbf{y}}_i \approx \mathbf{R}_n \mathbf{x}_{ni} + \mathbf{t}_n \tag{23}$$

where $\mathbf{x}_{ni}$ is the three-dimensional position of the ith atom in the nth ensemble member. To estimate $\overline{\mathbf{Y}}$ during EM we calculate the average structure:

$$\overline{\mathbf{y}}_i = \frac{1}{N} \sum_n (\mathbf{R}_n \mathbf{x}_{ni} + \mathbf{t}_n) \tag{24}$$

where N is the number of ensemble members. During Gibbs sampling, the coordinates of the average structure are generated by drawing random samples from Gaussians centered at the mean positions. To initialize both algorithms we calculate a mean structure using principal coordinate analysis [9, 10].

4.8 Generating random variates from the Generalized Inverse Gaussian distribution

The Gibbs sampler of the K distribution requires samples from the Generalized Inverse Gaussian (GIG) distribution [4]. To generate random variates from the GIG distribution defined as

$$GIG(x; a, b, p) = \frac{(a/b)^{p/2}}{2\, K_p(\sqrt{ab})}\, x^{p-1}\, e^{-\frac{ax+b/x}{2}} \tag{25}$$

with $a, b > 0$, $x \geq 0$ and p real, we first apply the following parameter transformation $y = \sqrt{a/b}\, x$ leading to:

$$p(y|\beta, p) = \frac{1}{2K_p(\beta)}\, y^{p-1} e^{-\beta(y+y^{-1})/2} \tag{26}$$

with $\beta = \sqrt{ab} > 0$. We only have to consider the case $p \geq 0$ because a transformation from y to y^{-1} changes the sign of p but leaves the form of the distribution (26) invariant. We now have:

$$y^{|p|-1} = \frac{y^{-3/2}}{\Gamma(|p|+1/2)} \int_0^\infty \mathrm{d}z \, z^{|p|-1/2} e^{-z/y}. \tag{27}$$

This representation suggests the following Gibbs sampling strategy for generating samples from distribution (26):

$$\begin{aligned} y &\sim IG(y; \beta, \beta + 2z) \\ z &\sim G(z; |p| + 1/2, y^{-1}) \end{aligned}$$

because the joint distribution $p(y, z|\beta, p) = IG(y; \beta, \beta + 2z)G(z; |p| + 1/2, 0)$ will have the GIG (26) as marginal distribution. The Inverse Gaussian distribution is a GIG for the special choice $p = -1/2$, the mean and variance are $\sqrt{b/a}$ and $\sqrt{b/a^3}$, respectively. To generate random samples from the Inverse Gaussian distribution IG is straightforward [11], as is the generation of a random variate from the Gamma distribution G. The complete algorithm is: (i) run the above Gibbs sampler for a number of iterations to produce a sample y (10 are sufficient in our experience); (ii) obtain a random sample from the GIG (25) by letting $\sqrt{b/a}\, y$ if $p \geq 0$ and $\sqrt{b/a}\, y^{-1}$ otherwise.

References

[1] Kelly L Damm and Heather A Carlson. Gaussian-weighted rmsd superposition of proteins: a structural comparison for flexible proteins and predicted protein structures. *Biophys J*, 90(12):4558–4573, Jun 2006.

[2] A. P. Dempster, N. M. Laird, and D. B. Rubin. Maximum likelihood from incomplete data via the EM algorithm (with discussion). *J. R. Stat. Soc. B*, 39:1–38, 1977.

[3] S. Geman and D. Geman. Stochastic Relaxation, Gibbs Distributions, and the Bayesian Restoration of Images. *IEEE Trans. PAMI*, 6(6):721–741, 1984.

[4] O. Barndorff-Nielsen. Exponentially decreasing distributions for the logarithm of particle size. *Proceedings of the Royal Society of London. Series A, Mathematical and Physical Sciences*, 353(1674):401–419, 1977.

[5] M. Habeck. Generation of three-dimensional random rotations in fitting and matching problems. *Computational Statistics*, 24:719–731, 2009.

[6] Nicholas J Higham. Matrix nearness problems and applications. In M. J. C. Gover and S. Barnett, editors, *Applications of Matrix Theory*, pages 1–27. Oxford University Press, 1989.

[7] W. R. Gilks and P. Wild. Adaptive rejection sampling for Gibbs sampling. *Applied Statistics*, 41(2):337–348, 1992.

[8] Thomas Minka. Estimating a gamma distribution. unpublished manuscript.

[9] J. Gower. Some distance properties of latent root and vector methods used in multivariate analysis. *Biometrika*, 53:325–338, 1966.

[10] S. G. Hyberts, M. S. Goldberg, T. F. Havel, and G. Wagner. The solution structure of eglin c based on measurements of many NOEs and coupling constants and its comparison with X-ray structures. *Protein Sci.*, 1:736–751, 1992.

[11] L. Devroye. *Non-uniform Random Variate Generation*. Springer Verlag, New York, 1986.
